# 免疫衰老在老年晚期非小细胞肺癌及其免疫治疗中的研究进展

**DOI:** 10.3779/j.issn.1009-3419.2025.102.28

**Published:** 2025-07-20

**Authors:** Na WANG, Yaning LUO, Haoyu LU, Siyuan CUI, Kui ZHAO, Fanming KONG

**Affiliations:** ^1^300381 天津，天津中医药大学第一附属医院; ^1^First Teaching Hospital of Tianjin University of Traditional Chinese Medicine, Tianjin 300381, China; ^2^300381 天津，国家中医针灸临床医学研究中心; ^2^National Clinical Research Center for Chinese Medicine Acupuncture and Moxibustion, Tianjin 300381, China; ^3^300381 天津，天津市中医肿瘤研究所; ^3^Tianjin Cancer Institute of Traditional Chinese Medicine, Tianjin 300381, China

**Keywords:** 肺肿瘤, 免疫衰老, 老年患者, 免疫治疗, Lung neoplasms, Immunosenescence, Elderly patients, Immunotherapy

## Abstract

肺癌是全球发病率和死亡率最高的恶性肿瘤之一，非小细胞肺癌（non-small cell lung cancer, NSCLC）是肺癌主要的病理类型之一。尤其在老年群体中，NSCLC仍然是肿瘤相关死亡的重要原因。随着全球人口老龄化的加剧，免疫衰老逐渐成为影响NSCLC发生、进展及免疫治疗效果的关键因素。免疫衰老是伴随年龄增长而发生的免疫系统功能衰退过程，表现为免疫细胞的数量和功能性改变，包括胸腺退化、T细胞耗竭、表观遗传改变、免疫反应减弱及慢性低度炎症状态等特征。本综述旨在综合分析免疫衰老在老年晚期NSCLC中的作用机制，并探讨通过干预免疫衰老过程来抑制NSCLC进展、改善免疫治疗效果的潜在治疗策略。

## Abstract (en)

Lung cancer remains the leading cause of cancer-related incidence and mortality worldwide. Among its histological subtypes, non-small cell lung cancer (NSCLC) accounts for the majority of cases, representing the predominant pathological type. Notably, in the elderly population, NSCLC continues to be a major contributor to cancer-related deaths. With the global ageing population, immunosenescence has emerged as a key factor influencing the occurrence, progression, and the efficacy of immunotherapy of NSCLC. Immunosenescence refers to the age-related decline in immune system function, which manifests as alterations in both the quantity and functionality of immune cells. These include thymic involution, T cell exhaustion, epigenetic modifications, weakened immune responses, and a chronic low-grade inflammatory state. This review comprehensively analyzes the role of immunosenescence in elderly patients with advanced NSCLC and proposes potential therapeutic strategies to intervene in the immunosenescence process. By targeting immunosenescence, these strategies aim to inhibit the progression of NSCLC and improve the effectiveness of immunotherapy.

随着全球人口老龄化的加剧，老年人群的肿瘤发病率呈持续上升趋势。2022年全球癌症数据^[1]^显示，超过2/3的肿瘤患者年龄在65岁以上。这一趋势在我国亦显著，尤其是在60岁以上的老年群体中，肺癌已成为男女患者中最常见的恶性肿瘤，其发病率和死亡率均居于首位^[2]^。非小细胞肺癌（non-small cell lung cancer, NSCLC）约占所有肺癌病例的85%，且由于早期症状不明显，大多数患者在确诊时已进入晚期，患者的5年生存率通常低于15%^[3]^。近年来，以程序性死亡受体1（programmed cell death 1, PD-1）及其配体（programmed cell death ligand 1, PD-L1）抑制剂为代表的免疫检查点抑制剂（immune checkpoint inhibitors, ICIs）在晚期NSCLC治疗中取得显著进展，晚期NSCLC患者的5年生存率提高至15.5%-23.2%，标志着免疫治疗在晚期NSCLC治疗领域中的突破^[4]^。尽管免疫治疗在晚期NSCLC的治疗中取得了良好的疗效和安全性，老年患者的免疫治疗仍面临诸多挑战。老年患者常伴有多种慢性基础病，免疫系统的衰退加剧了免疫相关不良事件的发生风险。老年患者的免疫反应较年轻患者更加复杂，可能导致免疫治疗的不良反应加重，甚至影响治疗的耐受性^[5]^。此外，由于临床试验中老年患者的比例较低，导致相关的数据和证据较为匮乏，这使得针对老年群体的治疗策略尚未完全明确^[6]^。因此，如何平衡免疫治疗的疗效与不良反应，优化老年NSCLC患者的免疫治疗方案，仍然是当前亟待解决的重要问题。

免疫衰老是衰老过程中免疫系统功能逐渐减退的表现，随着年龄的增长，免疫系统的功能和组成发生一系列可预测的变化。免疫衰老的标志具有多重生物学特征，涉及基因组、细胞、组织等多个层面，基因组不稳定性、端粒缩短 、表观遗传改变等14个免疫衰老标志相互交织，共同推动免疫系统的衰退^[7]^（图1）。识别这些关键标志为免疫衰老的干预和治疗提供了理论基础，对延缓免疫衰老、提高老年人群体免疫功能具有重要意义^[8]^。免疫衰老主要表现为免疫器官的退化和先天性、适应性免疫功能的障碍。首先，骨髓中的造血干细胞（hematopoietic stem cells, HSCs）随着年龄的增长，其分化能力逐渐减弱，导致免疫细胞功能的异常；胸腺逐渐萎缩，生成功能正常的T细胞的能力下降；同时，淋巴结组织发生重塑，生发中心数量减少^[9]^。其次，免疫细胞亚群的变化是免疫衰老的显著特征。先天性免疫细胞方面，树突状细胞（dendritic cells, DCs）对抗原的摄取能力降低，抗原加工与呈递功能也明显减弱；巨噬细胞趋向M2型转化，分泌免疫抑制因子；自然杀伤（natural killer, NK）细胞数量增多，但CD56^bright^NK细胞比例下降，且其细胞因子和趋化因子的生成以及穿孔素和颗粒酶的释放能力均显著降低；髓源性抑制细胞数量增加，且中性粒细胞的迁移功能受限，导致免疫细胞对免疫刺激的反应性降低，这使得老年患者对各类疾病的易感性增加^[10]^。适应性免疫细胞方面，B细胞的多样性减少，功能下降；T细胞数量减少，T细胞受体的多样性丧失，CD28受体的表达减少，同时调节性T细胞和记忆T细胞的数量增加，从而导致免疫系统对肿瘤的免疫监视和抑制作用减弱。此外，免疫衰老还伴随着慢性低度炎症的发生，衰老细胞通过分泌衰老相关分泌表型（senescence-associated secretory phenotype, SASP）因子，如血管生成因子、趋化因子和细胞因子，促进炎症反应的积累，并形成免疫抑制微环境^[11]^。这种慢性炎症状态降低了免疫反应的效率，使得肿瘤微环境（tumor microenvironment, TME）中免疫抑制细胞的招募增多，从而抑制正常免疫细胞的活性。尽管ICIs通过重新激活免疫细胞的功能，提升其抗肿瘤能力，但免疫衰老依然会对ICIs的治疗效果产生一定的影响。

**图1:**
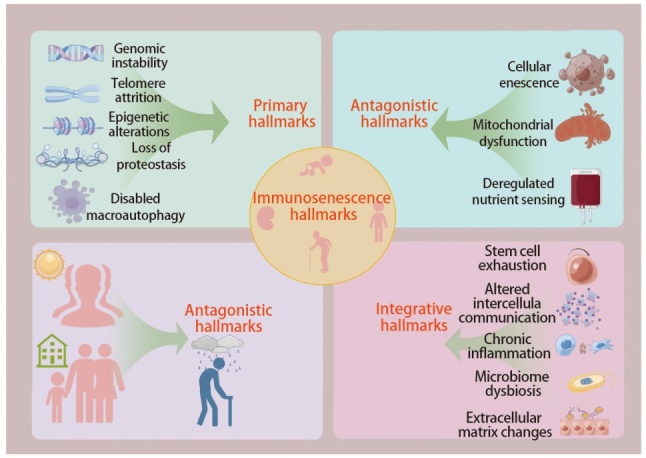
免疫衰老的14个重要标志，分为以下4类：（1）主要标志包括基因组不稳定性、端粒缩短、表观遗传改变、蛋白稳态丧失、巨自噬作用障碍；（2）拮抗标志包括细胞衰老、线粒体功能障碍、营养感应失调；（3）整合标志包括干细胞耗竭、细胞间通讯改变、慢性炎症、微生物群失调、细胞外基质变化；（4）心理社会隔离。

随着老龄化社会的到来，老年晚期NSCLC患者比例不断增加，使得研究免疫衰老与NSCLC免疫治疗的关系具有重要的临床意义。研究^[12]^表明，免疫衰老在老年人群中愈加严重，特别是在晚期NSCLC患者中，免疫系统功能的下降加重了肿瘤进展的风险。本综述结合免疫衰老的相关研究，提出了通过干预免疫衰老过程抑制NSCLC进展、改善NSCLC免疫治疗效果的潜在干预策略。

## 1 免疫衰老机制及其对老年NSCLC免疫治疗的影响

在肿瘤免疫治疗领域，ICIs如帕博利珠单抗（Pembrolizumab）和纳武利尤单抗（Nivolumab），已被广泛应用于NSCLC等多种恶性肿瘤的治疗。与此同时，免疫细胞治疗（adoptive cell therapy, ACT）和肿瘤疫苗等新兴治疗策略，为NSCLC患者提供了全新的治疗选择。ACT包括嵌合抗原受体T细胞疗法（chimeric antigen receptor T, CAR-T）、T细胞受体工程化T细胞疗法（T cell receptor-engineered T cell therapy, TCR-T）以及肿瘤浸润淋巴细胞疗法（tumor-infiltrating lymphocyte, TIL）等，这些方法通过重新编程免疫细胞，增强其识别和杀伤肿瘤细胞的能力^[13]^。然而，随着免疫衰老在老年NSCLC患者中作用的逐步揭示，免疫治疗在该人群中的疗效和安全性依然是当前研究的重点和难点（表1）^[14-25]^。免疫衰老不仅影响机体的免疫反应能力，还可能改变免疫治疗的效果，因此，探索免疫衰老对免疫治疗反应的具体影响，尤其是在老年NSCLC患者中的作用，已成为亟需解决的关键科学问题。

**表1:** 免疫治疗在老年晚期NSCLC患者中临床应用的研究数据

Clinical trial	Trial design	Population	Intervention	Primary endpoint	Key findings: OS (HR, 95%CI)
KEYNOTE- 010 (NCT01905657)^[14]^	Phase II/III, second or further line	Previously treated PD-L1 TPS≥1%	Pembrolizumab vs Docetaxel	OS, PFS	≥65 yr: 0.76 (0.57-1.02); <65 yr: 0.63 (0.50-0.79)
KEYNOTE- 024 (NCT02142738)^[15]^	Phase III, first line	Untreated PD-L1 TPS≥50%	Pembrolizumab vs chemotherapy	PFS	≥65 yr: 0.64 (0.42-0.98); <65 yr: 0.60 (0.38-0.96)
KEYNOTE- 042 (NCT02220894)^[16]^	Phase III, first line	Untreated PD-L1 TPS≥1%	Pembrolizumab vs chemotherapy	OS	≥65 yr: 0.82 (0.66-1.01); <65 yr: 0.81 (0.67-0.98)
CheckMate 017 (NCT01642004)^[17]^	Phase III, second line	Previously treated squamous	Nivolumab vs Docetaxel	OS	≥75 yr: 1.85 (0.76-4.51); 65-74 yr: 0.56 (0.34-0.91); <65 yr: 0.52 (0.35-0.75)
CheckMate 057 (NCT01673867)^[18]^	Phase III, second or third line	Previously treated nonsquamous	Nivolumab vs Docetaxel	OS	≥75 yr: 0.90 (0.43-1.87); 65-74 yr: 0.63 (0.45-0.89); <65 yr: 0.81 (0.62-1.04)
KEYNOTE- 189 (NCT02578680)^[19]^	Phase III, first line	Untreated nonsquamous	Chemotherapy+ Pembrolizumab or placebo	OS, PFS	≥65 yr: 0.64 (0.43-0.95); <65 yr: 0.43 (0.31-0.61)
KEYNOTE- 407 (NCT02775435)^[20]^	Phase III, first line	Untreated squamous	Chemotherapy+ Pembrolizumab or placebo	OS, PFS	≥65 yr: 0.74 (0.51-1.07); <65 yr: 0.52 (0.34-0.80)
IMpower 150 (NCT02366143)^[21]^	Phase III, first line	Untreated nonsquamous	Chemotherapy and Bevacizumab±Atezolizumab	OS, PFS	≥75 yr: 0.78 (0.51-1.19); 65-74 yr: 0.76 (0.56-1.00); <65 yr: 0.65 (0.51-0.82)
IMpower 130 (NCT02367781)^[22]^	Phase III, first line	Untreated nonsquamous	Chemotherapy±Atezolizumab	OS, PFS	≥65 yr: 0.78 (0.58-1.05); <65 yr: 0.79 (0.58-1.08)
IMpower 131 (NCT02367794)^[23]^	Phase III, first line	Untreated squamous	Chemotherapy±Atezolizumab	OS, PFS	≥75 yr: 0.81 (0.46-1.42); 65-74 yr: 0.87 (0.63-1.20); <65 yr: 0.91 (0.69-1.21)
CheckMate 227 (NCT02477826)^[24]^	Phase III, first line	Part 1A: PD-L1 TPS ≥1%; Part 1B: PD-L1 TPS <1%	Nivolumab+Ipilimumab vs Platinum-based chemotherapy	OS	≥75 yr: 0.85 (0.56-1.28); 65-74 yr: 0.75 (0.61-0.92); <65 yr: 0.71 (0.59-0.85)
CheckMate 9LA (NCT03215706)^[25]^	Phase III, first line	Untreated across all PD-L1 expression levels	2 cycles Platinum-based chemotherapy+Nivolumab+ Ipilimumab vs Platinum-based chemotherapy	OS	≥75 yr: 1.21 (0.69-2.12); 65-74 yr: 0.62 (0.46-0.85); <65 yr: 0.61 (0.47-0.80)

NSCLC: non-small cell lung cancer; OS: overall survival; PFS: progression-free survival; PD-L1: programmed cell death ligand 1; TPS: tumor proportion score; HR: hazard ratio.

免疫治疗通过解除免疫抑制、恢复T细胞的抗肿瘤功能，在老年NSCLC的治疗中已展现出显著疗效。然而，免疫衰老在老年NSCLC患者中普遍存在，并显著影响免疫治疗的效果。免疫衰老主要表现为免疫功能的衰退，特别是T细胞功能的减弱、免疫细胞浸润的减少及免疫耐受的增强，这些变化直接限制了免疫治疗在老年NSCLC患者中的临床疗效^[26]^。免疫衰老的机制复杂，涉及固有免疫与适应性免疫系统的双重衰退。在固有免疫方面，老年个体的NK细胞功能下降，DC细胞的活化能力减弱，导致免疫监视功能的减弱；在适应性免疫方面，尤其是CD8^+^ T细胞的分化与增殖能力下降，使得免疫反应显著减退。这些免疫功能的衰退使得免疫治疗在老年NSCLC患者中无法有效激活T细胞，未能充分恢复其对肿瘤的免疫监视和清除功能^[27]^。此外，免疫衰老还与TME的变化密切相关。老年NSCLC患者常伴有慢性低度炎症，为肿瘤免疫逃逸提供了有利条件，进一步削弱了免疫治疗的效果。

动物模型研究^[28]^表明，相较于年轻小鼠，老年小鼠在接受ICIs治疗后，治疗效果明显降低，这一现象初步揭示了年龄在肿瘤治疗反应中的潜在重要作用。首先，随着年龄增长，生理功能逐渐衰退，再加上多种共病的存在，直接影响了老年患者的治疗反应与耐受性。其次，老年患者群体普遍存在营养不良、认知障碍和功能下降等非特异性症状，这些问题不仅使治疗管理更加复杂，还可能导致老年患者在临床试验中的参与度减少^[29]^。由于这一群体在临床试验中的代表性不足，我们对年龄相关变化和免疫衰老如何具体影响免疫治疗效果的理解仍然十分有限，进而导致该群体缺乏有效的支持治疗数据。一项包含17项随机对照试验、共涉及10,291例患者的荟萃分析^[30]^结果显示，PD-1/PD-L1抑制剂能够显著延长75岁及以上老年NSCLC患者的总生存期。鉴于老年患者独特的生理和病理状态，迫切需要制定更加个性化的治疗策略，以减轻免疫衰老的不良后果。因此，深入研究老年NSCLC患者在免疫治疗过程中面临的具体挑战，合理设计针对该人群的特异性免疫治疗策略，对于有效提高治疗效果并最终提高患者生存质量具有重要意义。

## 2 靶向免疫衰老的治疗策略及其改善晚期老年NSCLC治疗效果的潜力

恢复免疫系统功能的策略是提升老年NSCLC患者抗肿瘤免疫反应的关键途径^[31]^。针对免疫衰老的干预措施应聚焦于重塑衰老免疫细胞功能，主要包括抗炎治疗和细胞因子疗法等方案。与此同时，生活方式的改变，尤其是增加适度的身体活动、调整膳食结构及药理学干预，可能成为延缓甚至逆转免疫衰老的有效策略。此外，衰老细胞清除药物的应用，作为一种新兴的治疗方法，通过靶向清除功能衰退的免疫细胞，也为减轻免疫衰老及其对肿瘤免疫治疗的不利影响提供了新的思路。

### 2.1 衰老细胞清除、促炎因子消除与免疫功能恢复

SASP严重影响免疫反应的有效性，因此针对衰老细胞的清除策略有望显著改善免疫治疗的效果。衰老细胞清除剂是一类能够选择性诱导衰老细胞凋亡的药物，通过去除衰老细胞从而缓解其对免疫系统及TME的负面影响^[32]^。其中，达沙替尼（Dasatinib）和槲皮素（Quercetin）作为著名的“返老药”，已在多种临床前研究^[33,34]^中显示出对包括肺癌在内的多种肿瘤的潜在治疗作用；这些药物通过特异性识别衰老细胞，最大限度地减少对健康细胞的损伤，使其成为肿瘤治疗中极具吸引力的候选药物。非诺贝特（Nicotinamide riboside）作为一种替代化合物，也被证明能够通过上调过氧化物酶体增殖物激活受体α（peroxisome proliferator-activated receptor alpha, PPARα）的表达，有效介导衰老细胞的选择性清除^[35]^。在KRAS驱动的肺部肿瘤模型中，巨噬细胞和内皮细胞是主要的衰老细胞群。研究^[36]^表明，清除衰老的巨噬细胞，特别是那些表达CSFR1或细胞周期抑制因子P16INK4A的巨噬细胞，可以显著降低肺部肿瘤负荷。另外，衰老细胞的清除还能够改善TME，通过免疫调节作用抑制肿瘤血管生成，从而对抗肿瘤生长和转移。在临床前模型中，衰老的巨噬细胞群的存在不仅在肺癌肿瘤中得到确认，也在肺部癌前病变的早期阶段被广泛观察到，这进一步强调了衰老细胞清除在肿瘤治疗中的潜在应用价值^[36]^。

此外，以B细胞淋巴瘤2（B-cell lymphoma 2, BCL-2）为代表的BCL蛋白家族，在细胞存活调节中发挥着至关重要的作用。研究^[37]^表明抑制BCL-2不仅能调节细胞存活，还对衰老细胞的清除具有潜在的影响。ABT-737作为一种BCL-2抑制剂，目前正被评估为潜在的抗衰老治疗药物，其在衰老细胞清除中的应用显示出良好的前景^[38]^。基于NK细胞的免疫治疗策略，已被证明能够有效清除衰老细胞并延长小鼠模型的寿命，同时表现出较好的安全性^[39]^。这一免疫疗法不仅能够显著降低衰老标志物的水平，还能减少SASP的产生且未观察到明显的毒性反应。这一发现为衰老细胞清除剂在抗衰老及肿瘤治疗中的应用提供了有力的支持。尽管衰老细胞清除剂在理论上具有广泛的应用前景，但它们的选择性仍然面临挑战。衰老细胞清除剂不仅靶向衰老细胞，还可能非选择性地作用于正常细胞，引发不良反应。因此，进一步的研究对于全面了解这些药物在临床中的安全性和有效性至关重要，尤其是在NSCLC等肿瘤的临床应用中，需评估其在不同免疫亚型患者中的疗效差异。

免疫衰老与慢性炎症的相互作用在肿瘤免疫逃逸过程中扮演着关键角色。促炎症细胞因子网络尤其是白细胞介素（interleukin, IL）-6及其相关信号通路，已被视为潜在的抗衰老治疗靶点^[40]^。IL-6抑制剂与其他信号通路抑制剂联合应用，在肺癌等肿瘤的免疫治疗中可以有效减轻免疫衰老对免疫细胞功能的负面影响，并增强免疫治疗的疗效^[41]^。此外，使用转化生长因子β（transforming growth factor beta, TGF-β）抑制剂，如TGF-β封闭抗体或TGF-β受体I激酶抑制剂，显示出改善免疫微环境、增强免疫细胞活性，进而提高肿瘤免疫治疗效果的潜力^[42]^。在临床转化中，如何精准调控TGF-β信号通路以增强肺癌患者的免疫治疗效果，将是未来研究的关键。

恢复免疫细胞功能是抗衰老免疫治疗的重要方向。通过外源性细胞因子的补充（如IL-2和IL-7），能够有效激活衰老免疫细胞，促进T细胞的增殖，并增强其识别和清除肿瘤细胞的能力^[43]^。尤其是在NSCLC的治疗中，恢复衰老免疫细胞的功能可以显著提高患者的免疫监视功能，从而抑制肿瘤生长和转移。然而，免疫细胞功能恢复的临床应用仍面临诸多挑战，例如免疫耐受性、治疗相关毒性等问题。

衰老细胞清除、促炎因子消除及免疫细胞功能恢复是当前免疫衰老治疗领域的研究热点，特别是在NSCLC治疗中的应用潜力巨大。然而，这些治疗策略在临床转化过程中仍面临诸多挑战，如治疗选择性的提高、免疫微环境的精准调控、治疗方案的个体化制定等。未来的研究应重点关注如何通过基础研究与临床数据的结合，进一步优化治疗策略，克服目前临床应用中的障碍。

### 2.2 免疫调节剂与免疫应答增强

调节衰老相关信号通路以逆转或避免衰老相关表型的策略，已成为免疫衰老干预的一个重要方向。漆黄素（Fisetin）通过抑制促炎途径，例如信号传导及转录激活蛋白3（signal transducer and activator of transcription 3, STAT3）或核因子κB（nuclear factor kappa-B, NF-κB），能够有效干预衰老特征并逆转TME中的免疫抑制^[44]^。雷帕霉素（Rapamycin）作为一种经典的哺乳动物雷帕霉素靶蛋白（mammalian target of rapamycin, mTOR）抑制剂，已被广泛研究并证实能够破坏衰老环境中的核因子E2相关因子2（nuclear factor erythroid 2-related factor 2, Nrf2）通路，从而有效调节与衰老相关的免疫反应。Nrf2是调节细胞抗氧化应激反应的重要转录因子，mTOR抑制剂通过抑制Nrf2通路，帮助恢复免疫系统的功能，减缓免疫衰老的进程^[45]^。此外，靶向腺苷酸激活蛋白激酶（AMP-activated protein kinase, AMK）信号通路的治疗策略为免疫衰老的干预提供了新的视角。通过丝裂原活化的细胞外信号调节激酶（mitogen-activated extracellular signal-regulated kinase, MEK）途径调节衰老T细胞的功能，研究^[11,46]^表明这一策略能够有效恢复免疫细胞的功能，改善衰老免疫系统的效能。这些研究成果证明了通过靶向不同分子通路来抑制衰老过程、改善免疫功能的潜力。但在临床应用中，如何针对特定免疫亚群体实施精准治疗，仍是未来的研究重点。特别是对于NSCLC患者，由于免疫微环境的复杂性，信号通路的靶向调节可能需要与其他免疫治疗手段联合使用，以增强治疗效果。

此外，肿瘤疫苗是一类重要的免疫增强剂治疗策略，涵盖了肿瘤抗原相关疫苗、新抗原相关疫苗以及基于细胞的疫苗，通过主动免疫机制激发和增强机体对肿瘤的特异性T细胞免疫反应^[47]^。肿瘤抗原相关疫苗通过靶向肿瘤特异性抗原或肿瘤相关抗原，能够有效激活特异性T细胞，从而促进免疫系统识别并清除肿瘤细胞。对于NSCLC，靶向肺癌特有抗原[如表皮生长因子受体（epidermal growth factor receptor, *EGFR*）突变或Kirsten大鼠肉瘤2型病毒癌基因同源物（Kirsten rat sarcoma viral oncogene homolog, *KRAS*）突变]疫苗的研究取得了初步进展，但如何突破抗肿瘤免疫逃逸和肿瘤异质性问题，仍是临床转化的难题^[48]^。新抗原疫苗则通过靶向肿瘤细胞内新表位（neoantigens）的免疫反应，进一步提高治疗的特异性和疗效。新抗原是肿瘤细胞特有的突变产物，这些突变使得肿瘤细胞与正常细胞区别开来，成为理想的疫苗靶点。在NSCLC中，基于新抗原的个性化疫苗已在临床前模型中展现出良好的抗肿瘤效果，但如何在临床中进行新抗原的精准筛选和疫苗的快速生产，仍需要解决。基于细胞的疫苗则通过外源性细胞处理与肿瘤细胞的抗原呈递相结合，激活免疫系统的抗肿瘤反应。通过对患者免疫细胞的体外处理，使其能够更好地识别肿瘤细胞，并在体内对肿瘤进行有效的免疫清除。该策略不仅增强了免疫系统的抗肿瘤功能，而且有助于改善TME中的免疫逃逸现象^[49]^。对于NSCLC患者，基于细胞的疫苗可能通过重新激活患者自身的免疫系统，帮助突破肿瘤的免疫抑制屏障。临床研究正逐步深入，但细胞处理的复杂性和患者个体差异依然是临床应用中的挑战。

### 2.3 个性化免疫治疗与新兴精准干预策略

CAR-T细胞疗法在靶向衰老肿瘤细胞方面展现出良好的潜力，Amor等^[50]^研究表明，使用尿激酶型纤溶酶原激活物受体（urokinase plasminogen activator receptor, uPAR）特异性CAR-T细胞治疗的小鼠模型中，衰老的肿瘤细胞数量显著减少，为其在老年肿瘤患者中的应用提供了理论依据。尽管CAR-T疗法在治疗血液肿瘤方面取得了突破性进展，但其在实体瘤，特别是在晚期老年NSCLC中的应用仍面临许多挑战。一项涵盖30项临床试验、共541例接受CD19 CAR-T治疗的患者数据分析^[51]^显示，年轻患者（<44岁）治疗后的完全缓解率明显高于中年（45-59岁）和老年患者（>60岁）。这一结果提示，年龄可能是影响CAR-T疗法抗肿瘤效果的重要因素。老年NSCLC患者普遍存在免疫功能衰退和免疫抑制微环境，T细胞活性降低，这些因素可能削弱CAR-T细胞的抗肿瘤效果。

纳米技术的快速发展为靶向衰老细胞治疗提供了新的策略。纳米颗粒因其可调控的理化性质、高度的靶向性以及良好的生物相容性，被广泛应用于肿瘤治疗和衰老干预研究中^[52]^。针对免疫衰老相关的TME，研究者已设计出专门用于识别和清除衰老细胞的智能纳米颗粒系统。其中，一类共载阿霉素（Doxorubicin）与口服BCL-2抑制剂Navitoclax的功能化纳米颗粒——GalNP（Dox）与GalNP（Nav），已被开发用于构建高选择性的抗衰老纳米治疗系统^[53]^。这些纳米颗粒通过β-D-半乳糖基配体靶向衰老细胞表面特有的β-半乳糖苷酶，经过内吞作用后与溶酶体囊泡融合，精准释放药物诱导衰老细胞凋亡，且几乎不影响正常健康细胞。尽管该纳米系统在NSCLC等衰老模型中表现出良好的选择性与安全性，但在临床应用中如何进一步优化纳米颗粒的靶向性与释放机制，减少非靶向细胞的损伤，仍然是未来研究的关键。

HSCs衰老被广泛认为是驱动免疫衰老的关键病理基础。随着年龄的增长，HSCs功能逐渐退化，其分化倾向发生改变，逐步偏向髓系谱系，同时其分化生成的淋巴系细胞数量与功能均显著下降，最终导致免疫系统整体反应能力削弱^[54]^。慢性炎症作为加速HSCs衰老的重要诱因，通过损伤DNA修复机制和自我更新能力，进一步推动HSCs功能耗竭。Ross等^[55]^通过以HSCs为治疗靶点，选择性地清除具有髓系偏向特征的my-HSCs（myeloid-biased HSCs），研究观察到淋巴祖细胞、幼稚T细胞与B细胞的产量显著上升，同时与免疫系统功能障碍相关的标志物水平下降，从而在老年小鼠中有效逆转了免疫衰老现象。此外，Netrin-1蛋白作为一种在骨髓微环境中关键表达的因子，被证实能够激活HSCs的DNA损伤应答途径，恢复其干性状态和分化潜能。研究^[56]^表明，通过外源性补充Netrin-1，可显著提升衰老HSCs的功能活性，有望重建老年宿主的免疫能力。在老年NSCLC患者中，通过清除衰老的HSCs亚群、修复TME和激活其干性，或可恢复造血系统的稳态平衡，提升免疫细胞数量与质量，进而增强抗肿瘤免疫应答。

干细胞治疗作为一种利用干细胞再生潜能修复或替代功能受损免疫细胞的治疗策略，已成为应对年龄相关病理疾病的重要手段^[57]^。随着干细胞在免疫系统中的重要作用逐步得到认识，利用干细胞进行免疫衰老干预，已被提出作为提高老年人免疫功能、改善疾病预后的潜在治疗方法。器官移植，作为一种典型的干细胞治疗应用，已被证明能够显著缓解年龄相关的衰退性损伤，并对抗衰老进程。近年来，胸腺再生手术作为一种干细胞治疗应用，已显示出显著的免疫功能恢复效果。该手术通过促进胸腺再生，恢复T细胞的生成与功能，改善老年个体的免疫反应并对抗衰老进程。研究^[58]^表明，胸腺再生不仅改善了免疫功能，还逆转了与年龄相关的疾病风险指标，并导致生物学年龄的逆转。然而，胸腺再生手术的临床应用仍面临挑战，特别是在器官来源问题和排斥反应的控制方面^[59]^。此外，随着免疫干预研究的深入，未来可能通过干细胞移植、免疫细胞重建等方法，进一步提升老年NSCLC患者的免疫功能，为免疫治疗提供新的支持。

综上所述，免疫衰老是一个多因素驱动的复杂生理过程，涉及多条信号通路的交互作用以及不同细胞类型间的动态调控。尽管当前的免疫治疗取得了一定进展，但试图通过非特异性方式全面激活免疫系统往往导致免疫失衡，甚至可能引发自身免疫相关并发症。因此，识别并靶向调控在免疫衰老中起关键作用的分子机制，成为安全有效重塑老年人免疫功能的核心策略之一。近年来，针对衰老免疫细胞中异常活化或抑制的信号通路，如p38 MAPK、NF-κB、mTOR、JAK/STAT等，开发的靶向药物和小分子干预措施展现出良好的前景。这些干预不仅有助于从分子水平逆转免疫细胞的功能衰退，还可能增强免疫系统对肿瘤抗原的识别和清除能力，从而提升免疫监视功能。然而，当前这些策略在老年NSCLC患者中的临床应用仍面临挑战。由于老年NSCLC患者普遍存在免疫应答受限的情况，传统免疫治疗的疗效往往不理想。因此，靶向免疫衰老的治疗策略，尤其是与ICIs等治疗手段的联合应用，可能为这一特殊群体提供突破性的治疗效果，并表现出显著的协同增效作用^[60]^。虽然靶向免疫衰老的治疗策略为老年NSCLC患者提供了新的治疗思路，但其临床应用的普及仍需更多的高质量循证数据支持。通过多中心、随机对照临床试验的推进，结合精准时机、联合治疗和个体差异化研究，靶向免疫衰老策略有望为老年NSCLC患者提供突破性疗效，并最终推动个性化免疫治疗的临床广泛应用。

## 3 结语

随着全球人口老龄化的加剧，免疫衰老对NSCLC发病机制及治疗应答的影响日益显著。免疫衰老表现为T细胞持续耗竭、NK细胞功能下降及慢性低度炎症等特征，这些免疫功能障碍广泛存在于老年NSCLC患者中，严重削弱了其对抗肿瘤治疗的应答能力。因此，针对免疫衰老的干预策略是改善这一群体治疗效果的关键。当前的研究集中在以下3类治疗策略：一是通过清除衰老细胞来改善免疫状态，消除功能障碍免疫细胞及其促炎因子；二是利用免疫调节剂恢复免疫应答效能；三是制定个性化免疫治疗方案，依据患者的衰老免疫表型和分子特征进行精准干预。然而，将这些策略转化为临床应用仍面临挑战，主要包括免疫衰老的个体差异性、靶点筛选和生物标志物验证不足以及潜在的免疫过激反应和副作用风险。未来的研究应聚焦于以下几个方向：首先，开展免疫衰老的精准评估方法，筛选更为特异的生物标志物，以便对患者免疫状态进行有效监测；其次，探索多策略联合治疗，以减轻副反应并提高疗效；最后，加强跨学科合作，整合免疫学、肿瘤学和老年医学等领域，为个体化免疫治疗方案的优化提供更加坚实的理论基础。
